# Anticipated nursing care as perceived by nursing students: Findings from a qualitative study

**DOI:** 10.1002/nop2.883

**Published:** 2021-05-03

**Authors:** Lisa Lunardelli, Matteo Danielis, Michela Bottega, Alvisa Palese

**Affiliations:** ^1^ Department of Medical Sciences Udine University Udine Italy; ^2^ Department of Biomedicine and Prevention Tor Vergata University Rome Italy

**Keywords:** anticipated nursing care, nursing education, nursing practice, nursing students, qualitative study

## Abstract

**Aim:**

To explore the perceptions of nursing students on the phenomenon of anticipated nursing care.

**Design:**

A descriptive‐qualitative study was performed in 2019 according to the Consolidated Criteria for Reporting Qualitative Research principles.

**Methods:**

Data were collected using 16 face‐to‐face, audio‐recorded interviews across four Italian Bachelor of Nursing degrees. Then, content analysis was performed, identifying, analysing and describing the anticipated nursing care phenomenon as perceived by nursing students.

**Results:**

Administering medications, providing fundamentals of care, managing some clinical procedures, freeing up the patient's bed and starting the shifts early emerged as the most anticipated nursing interventions. Stable, older patients who were more functionally dependent were reported to receive some fundamental nursing care before the expected time, while older, stable and more independent patients were used to receiving medications in advance. Anticipated nursing care is triggered by factors at the time management, resource, programming, professional and organizational levels.

## 1 INTRODUCTION

During their clinical rotations, nursing students are exposed to real situations, and, alongside good examples of clinical practice, they might also witness examples of poor practice that may threaten patient safety (Ion et al., 2015). Among several experienced nursing practices, missed nursing care (MNC), or any aspect of required nursing care that is omitted (in part or in whole) or delayed (Kalisch, 2006) – also known as care left undone, rationing of nursing care and unfinished nursing care (Bassi et al., 2018) – which leads to negative outcomes (Jones et al., 2019) might be witnessed by nursing students.

As documented, students have developed a progressive awareness that MNC exists as an expression of the gap between theory and practice (Greenway et al., 2019). It has also been reported that students have learned that no nurse would intentionally miss nursing care, but sometimes this can happen due to many competitive interventions and priorities (Gibbon & Crane, 2018). In witnessing MNC episodes, students experienced a cognitive dissonance threatening their professional and personal values (Bagnasco et al., 2017) and felt negative emotions (Gibbon & Crane, 2018).

At the same time, however, nursing students struggle to express criticism because this can affect their internship evaluations (Ion et al., 2015). At the end of the complex process of continued exposure to MNC, students have reported to live in a sort of “culture of acceptance” as a process of professional socialization and “pragmatic acceptance” in which MNC progressively becomes an integral part of the working reality (Gibbon & Crane, 2018). However, students have been found somewhat unaware and uninformed on the reasons for MNC, as they are not fully involved in the decision‐making processes (Gibbon & Crane, 2018).

## 2 BACKGROUND

### 2.1 Anticipated nursing care and nursing students

Alongside MNC, students are also exposed to anticipated nursing care (ANC), which has been defined as care required by patients that is delivered early (in part or in whole) compared to when it is expected by patients and specified by the nursing care plan (Bottega & Palese, 2020). ANC is completely the opposite of MNC: it refers to any intervention delivered in an anticipated manner on a regular basis rather than occasional or in critical circumstances. Moreover, the process of “moving forward” – in nurses’ words – is not a result of a request of anticipation by patients or other healthcare professionals. More accurately, ANC is triggered by some reasons at both the nurse (e.g. the desire to be a well‐performing nurse) and unit levels (e.g. variations in the complexity of patients or in the nursing workloads); some reasons reflect those already reported for MNC (Kalisch & Xie, 2014).

As reported mainly in anecdotical examples and a small amount of empirical data (Greenway et al., 2019; McLeod et al., 2015; Palese, 2012; Rytterstrom et al., 2011), clinical nurses tend to anticipate (instead of postponing or omitting) some nursing interventions. Examples include night nurses waking patients early to finish all activities by the end of their shift, providing hygiene care early in the morning or preparing patients for the night by doing these prep activities before breakfast and just after dinner, respectively; and preparing medications in advance by leaving them on bedside tables as well as starting with the administration of medications before the prescribed time (e.g. administering medicines prescribed for 4 p.m. at 3 p.m. or earlier) (Palese, 2012; Rytterstrom et al., 2011). More recently, it was shown that the majority of anticipated interventions might also be delayed and/or missed, confirming this as a potential source of inequity among patients cared for by nurses (Bottega & Palese, 2020).

Anticipating some interventions can lead to negative outcomes: for instance, sleep patterns can be disturbed, and the pharmacodynamics of some medications can be altered (e.g. in the case of Parkinson's disease, according to anecdotal data provided). Moreover, threatening the patient's daily rhythms is against the clinical nurses’ aims of addressing and facilitating the patient's fundamental needs (Richards et al., 2018). As nursing students are inclined to repeat their preceptors’ patterns of behaviour, the phenomena that ultimately precede compromised nursing care should be explored also in undergraduate education. Despite this, the emerging concept of ANC has never been studied from the perspective of nursing students.

### 2.2 Rationale and aim of the study

The aim of this study is to contribute to the understanding of nursing students’ perceptions on the ANC phenomenon to identify strategies for enhancing the ability of the future workforce to deliver interventions when expected and, ultimately, to improve their priority‐setting skills, thus minimising delayed, missed and anticipated care, which can all lead to negative outcomes. Hence, the aim of this study was to explore the perceptions of nursing students on the phenomenon of ANC as they experienced it during their clinical rotations.

## 3 METHODS

### 3.1 Study design

A descriptive‐qualitative study (Sandelowski, 2010) was performed in 2019. The Consolidated Criteria for Reporting Qualitative Research (COREQ) (Tong et al., 2007) were followed in reporting the methods and the findings (Table S1).

### 3.2 Setting and participants

To include a wide range of students with different clinical experiences, the study involved four Bachelor of Nursing degree programmes, each three years in length, located in Northeast Italy and with an average of 300 students each. Each nursing programme was contacted, and appropriate authorization was obtained for the study. Then, a faculty member responsible for clinical placement was approached and briefed on the inclusion criteria: s/he was free to identify which students to involve and was responsible for the initial contact to present the general aim of the study, to request their authorization, and to give their email contact to the study coordinator.

A purposive sample (Patton, 2015) of nursing students was employed. Students, who were approached and invited to participate, (a) were regularly attending a nursing programme as students of the first, second or third year of nursing education; (b) attended their clinical rotations for at least four weeks in medical and/or surgical wards; (c) obtained a positive mark in the competences achieved; and (d) gave their written consent. Thus, students who (a) attended their nursing education discontinuously (e.g. due to health issues, work commitments or commitments abroad), (b) spent less than three weeks of clinical learning experience in the same unit and (c) reported a negative evaluation in the competences achieved, which could influence their understanding of the nursing care delivered in the unit, were excluded.

A total of 18 students were first identified; 16 were contacted and agreed to participate in the study, and two students refused to participate because of their limited time due to work responsibilities during nursing education. Most of the students interviewed were attending the third year of nursing education. Their average age was 23.1 years (standard deviation [*SD*] 4.06), and the majority (12/16:75%) were female (Table 1). At the time of the study, they were attending their clinical rotations mainly in acute care settings and in secondary care.

**TABLE 1:** Participants profile

NS	Nursing programme^a^	Age, years	Gender	Academic year attended	The most recent clinical rotation experience	Setting	Interview duration, minutes:seconds
1	1	20–22	Female	3	Intensive Care Unit	Acute Hospital	27:24
2	1	20–22	Male	3	Medical Unit	Acute Hospital	26:57
3	2	20–22	Male	3	Surgical Unit	Academic Hospital	27:22
4	2	26–28	Male	3	Emergency Department	Academic Hospital	39:48
5	2	23–25	Female	3	Neurology Unit	Academic Hospital	27:17
6	2	20–22	Female	3	Cardiosurgical Intensive Care Unit	Academic Hospital	21:05
7	2	20–22	Female	2	Rehabilitation Unit	Rehabilitation Hospital	30:49
8	2	20–22	Female	3	Medical Unit	Academic Hospital	29:13
9	1	20–22	Female	2	Surgical Unit	Acute Hospital	20:00
10	1	29–31	Female	1	Pneumology & Nephrology Units	Acute Hospital	16:36
11	1	20–22	Female	1	Surgical Unit	Acute Hospital	24:52
12	1	20–22	Female	2	Medical Unit	Acute Hospital	33:54
13	2	20–22	Female	2	Medical Unit	Academic Hospital	32:59
14	2	20–22	Male	3	Surgical Unit	Academic Hospital	40:58
15	3^b^	35–37	Female	3	Medical Unit	Academic Hospital	49:52
16	4^b^	20–22	Female	3	Coronary Intensive Care Unit	Acute Hospital	43:05

Abbreviation: NS, Nursing Student.

^a^ Nursing Programme no. 1, 2, 3, or 4.

^b^ Interviewed via Skype.

### 3.3 Data collection

Data were collected using face‐to‐face, audio‐recorded interviews performed from July to September 2019. The interview guide was developed according to the open‐ended questions reported in Table 2 and the evidence available on ANC (Bottega & Palese, 2020). A preliminary piloting of the interview guide with one interview was conducted by two researchers (refer authors), and it was subsequently confirmed that no changes were requested. Because of the descriptive nature of the study (Sandelowski, 2010), in‐the‐field notes were not collected.

**TABLE 2:** Semi‐structured interview question guide

General data: gender, age, academic year attended (first, second, or third), the clinical learning experience with which the interview is most concerned; the unit and the setting attended
Questions:
1. During your clinical experience, have you experienced or witnessed occasions where nursing care interventions have been delivered early as compared to the expected time?
2. Do you remember, and can you list the nursing interventions that you experienced or witnessed which were anticipated most frequently?
3. Can you remember or speculate on the reasons why these interventions were anticipated? According to your experience, can you remember and describe who took the initiative to anticipate the nursing interventions? What was your role as a nursing student with regard to these interventions?
4. Have you experienced or witnessed some patients or conditions (e.g., shifts) where interventions have been delivered more often in an anticipated manner than specified? Can you describe in detail your experience…
5. What effects did you observe/perceive when nursing interventions were anticipated?
6. Please feel free to share other elements of this experience…

Each eligible student was approached by the senior researcher (refer authors), who explained the study aims and procedures. Then, after written informed consent was obtained, interviews were conducted in individual sessions in locations chosen by the students, such as in places associated with the nursing programme (e.g. the campus or library) and outside (e.g. home); in addition, two interviews were performed via Skype in accordance with the preference of the student due to distance (>100 miles). During the first two interviews, two researchers (refer authors) were present to provide supervision to the junior researcher. Subsequently, only one researcher conducted the interviews. The average duration of the interviews was 30:45 min and ranged from 16:36–49:52. When saturation was achieved (Vasileiou et al., 2018) on the basis of the verbatim transcribed interviews – as judged by two researchers independently – the recruitment of participants ended. Two transcribed interviews were reported to participants for comments and corrections, making the first member checking (Sandelowski, 2010), thus also ensuring the quality of the process.

### 3.4 Data analysis

Researchers were interested in learning about ANC as experienced by nursing students. Therefore, analysing their responses as manifested forms of beliefs and constructs was defined as the intent of the data analysis (Smith & Eatough, 2006). Initially, researchers shared with each other their preconceptions regarding ANC (Vaismoradi et al., 2013) and its potential meaning for students; this was intended to prevent data analysis biases. Then, transcribed interviews were numbered (interviewed nursing student no. 5, NS 5) and were read and reread (Vaismoradi et al., 2013) by two researchers who performed the data analysis; by rereading the transcripts several times independently; they obtained a global view and developed familiarization with the data. Next, a content analysis (Vaismoradi et al., 2013) was performed by identifying, analysing and reporting the phenomenon as perceived by nursing students. By rereading the transcripts, researchers identified meaningful data independently and then agreed upon them (Sandelowski, 2010). Data with similar content were integrated and organized in accordance with the aims of the study, as follows: (a) the phenomenon of ANC as experienced by nursing students during their clinical rotations, (b) its antecedents and (c) consequences according to the students. The meaning of each unit was described, and quotes were integrated. Strategies used to ensure rigour and trustworthiness were performed and are summarized in Table 3.

**TABLE 3:** Strategies to ensure rigour and trustworthiness

(a) Data collection: ‐ Two pilot interviews were performed to assess whether the format of the interview guide was clear, comprehensible, and feasible: no changes were needed. ‐ Two researchers performed the interviews, and both were female; (a) one was a nursing student at the end of her education (LL, see authors) and (b) the second was a senior PhD researcher (AP, see authors), expert both in research methodologies and in conducting interviews. The senior supervised the junior researcher in the first two interviews as the latter was competent in conducting clinical interviews but not in conducting research interviews. Both researchers introduced themselves to each participant student, and stated their current position and the study aims and procedures. ‐ A nursing student conducted the interviews with the intent to ensure the maximum degree of freedom and to avoid any pressure from educational authorities: actively involving nursing students, and making them responsible for the learning process of peers has been documented as effective in order to improve their confidence with nursing research. ‐ It was ensured that the interviewer and each participant were not classmates in previous or current clinical placements (Tong et al., 2007). ‐ At the end of each taped interview, it was immediately transcribed verbatim in order to share the contents with the senior and the junior researcher who progressively assessed data saturation (Vaismoradi et al., 2013).
(b) Data Analysis: ‐ Member checking of the transcripts was performed (Morse, 2015) to ensure accuracy in the transcribing process. ‐ Two researchers performed the entire process independently; at each step, they shared and agreed upon the findings ensuring inter‐rater reliability and triangulation during the data analysis (Morse, 2015). ‐ At the end, the consistency of the analysis was ensured through cross‐checking the findings which emerged and by additionally reading quotes extracted from the interviews (Morse, 2015). ‐ Data dependability was also ensured by providing a detailed data analysis and direct quotations appropriately numbered (e.g., NS1).

Abbreviations: NS, Nursing Student; NS1, Nursing Student interview number 1.

### 3.5 Ethical issues

Authorization to approach students was obtained from the dean of the nursing programmes involved by presenting the research protocol and ensuring that the project would be conducted in accordance with international ethical principles. According to Italian laws and the nature of the study, no formal authorization from an ethical committee was required. Before the interview, eligible nursing students were informed of the study aims, and their written informed consent was obtained. Anonymity was ensured at the individual and the nursing programme levels. Moreover, examples reported during the interviews by nursing students were all anonymized in their contextual features as well as their age, which is reported in ranges to protect students. Furthermore, students were free to withdraw from the interview at any time, and those who participated received no rewards.

## 4 RESULTS

**FIGURE 1:**
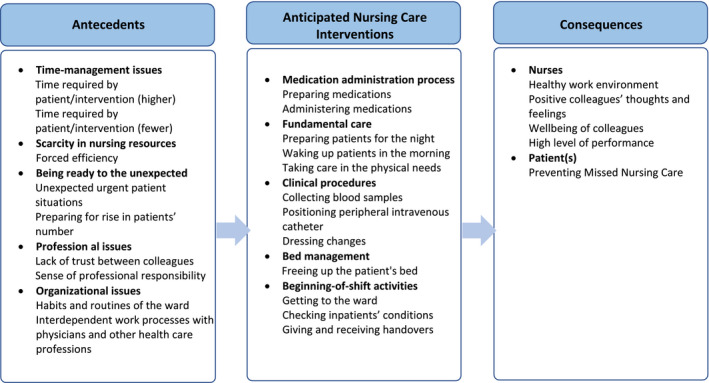
The Anticipated Nursing Care phenomenon as perceived by nursing students

According to the experiences of nursing students, ANC refers to a particular phenomenon where specific interventions are carried out prematurely, due to various reasons (antecedents) and leading to consequences, which are summarized in Table 4 and Figure 1.

**TABLE 4:** Anticipated nursing care interventions: categories, codes and quotes

Categories	Codes	Quotes	Nursing students
Anticipated nursing care interventions			
Medication administration process	Preparing medications	'*nurses moved up both with the preparation and the administration and the medications therapy … by preparing them in the morning, all doses that should be administered up to 2 p.m. and the same for the afternoon*' (NS6). '*nurses* *moved up both with the preparation and the administration and the medications*' (NS6). '*I’m referring when there are more of two closed medications … as for example at 8 a.m. and 10 a.m. these are administered together*' (NS16)	NS1, NS2, NS3, NS4, NS5, NS6, NS7, NS8, NS9, NS10, NS11, NS12, NS13, NS14, NS15, NS16
	Administering medications	'*I’m referring when there are more of two closed medications … as for example at 8 a.m. and 10 a.m. these are administered together'* (NS16)	
Fundamental care	Preparing patients for the night	*‘relatives* *left the unit at 7 p.m., … and all patients are prepared for the night early’* (NS14)	NS2, NS3, NS4, NS5, NS6, NS7, NS8, NS9, NS10, NS11, NS13, NS14, NS15
	Waking up patients in the morning	*‘we are used to waking up the patient to do some activities, for example: blood samples, body temperature’* (NS2)	
	Taking care of physical needs	*‘hygiene* *and mobilisation … they tend to do early’* (NS3)	
Clinical procedures	Collecting blood samples	*‘I have been through to move up the blood sample at the end of the night shift’* (NS6)	NS1, NS2, NS6, NS7, NS9, NS10, NS13, NS14, NS15, NS3, NS4, NS16
	Positioning peripheral intravenous catheter	*‘[peripheral venous catheter] yes, sometimes we applied it just when the patient entered in the ward’* (NS13). *[the* *second peripheral venous catheter] … it is inserted second when the first has some signs of phlebitis*' (NS3)	
	Dressing changes	*‘yes, it can happen before the expected time, for example when the deadline is the following morning’* (NS16)	
Bed management	Freeing up the patient's bed	*‘[discharge]it happens, patients are there without their bed’* (NS7)	NS3, NS6, NS7, NS8, NS13, NS15
Beginning‐of‐shift activities	Getting to the ward	*‘nurses* *are used to arriving much earlier than expected’* (NS4)	NS1, NS2, NS3, NS4, NS6, NS7, NS9, NS12, NS13, NS14, NS15, NS16
	Checking inpatients’ conditions	*‘he* *looks at patients, those who are still hospitalised, and those who are new, he is starting to look at the record’* (NS1)	
	Giving and receiving handovers	*‘[they arrive early at shift start] to start handover sooner’* (NS2)	
Antecedents			
Time management issues	Time required by patient/interventions (higher)	*‘they can ask for more time, thus* *we try to perform it before’* (NS14)	NS1, NS2, NS4, NS6, NS9, NS10, NS13, NS16
	Time required by patient/interventions (fewer)	*‘we* *do it first because you know it takes a short time’* (NS4)	
Scarcity in nursing resources	Forced efficiency	*‘is* *all modelled according to the organisation and ward resources’* (NS3; NS4; NS6; NS8)	NS2, NS3, NS5, NS6, NS8, NS9, NS10, NS11, NS13, NS14, NS16
Being ready for the unexpected	Unexpected urgent patient situations	*‘with certain nurses they used to do everything as soon as possible so that, as sometimes happens, a hospitalisation arrives for instance, we are free’* (NS16)	NS1, NS2, NS3, NS4, NS6, NS7, NS8, NS9, NS10, NS12, NS13, NS14, NS16
	Preparing for a rise in patient numbers	*‘you* *know that if the ward is full … you cannot give to the new patient a bed’* (NS6)	
Professional issues	Lack of trust between colleagues	*‘because sometimes you don't trust colleagues, you don't* *trust them, so you prefer to do something without leaving it to them’* (NS1)	NS1, NS4, NS6, NS7, NS8, NS16
	Sense of professional responsibility	*‘as* *nurses we have to perform as much, we can’* (NS16)	
Organisational issues	Habits and routines of the ward	*‘ward* *needs’* (NS7)	NS1, NS2, NS3, NS4, NS5, NS6, NS7, NS8, NS9, NS10, NS12, NS13, NS14, NS15, NS16
	Interdependent work processes with physicians and other health care professions	*‘we* *had to do things in advance because the physician had to have the medical chart’* (NS12)	
Consequences			
Nurses	Healthy work environment	*‘there* *was always a tendency to move up to avoid delays or extra workloads to colleagues that will arrive on the next shift’* (NS10)	NS1, NS2, NS5, NS7, NS8, NS9, NS10, NS11, NS12, NS13, NS14, NS16
	Positive colleagues’ thoughts and feelings	*‘solidarity’* (NS14)	
	Well‐being of colleagues	*‘finish* *the shift having completed everything’* (NS6)	
	High level of performance	*‘in* *order when they come to take care of patients’* (NS8)	
Patient(s)	Preventing missed nursing care	*‘to* *the last patient you arrive with half hour of delay and so you can miss some interventions’* (NS3)	NS1, NS3, NS4, NS5, NS6, NS13

Abbreviations: NS, Nursing Student; NS1, Nursing Student interview number 1.

### 4.1 The ANC phenomenon as perceived by nursing students

Students reported that performing interventions before the expected time was experienced in all units where they attended their clinical rotation(s): this has been reported as routine, in an attempt to “move forward” (NS1). ANC has been defined as a “road map” (NS2), “a philosophy of nursing work” (NS7), a “mentality” (NS8) or a “rule” (NS9) of “the sooner you do it, the better it is” (NS11) and

It was experienced in all wards (NS1)

Students distinguished ANC from proactive nursing care, where nurses perform something before the time expected to prevent issues, to ensure comfort or to anticipate a patient's need:

We went to the patient assuming he was tired, and then we moved him from the wheelchair to the bed … this is not doing something prematurely (NS4)

Two groups of patients were reported to be at risk of receiving ANC: stable, older patients more dependent on nursing care, such as patients with cognitive decline, who are used to receiving basic nursing care early:

With older patients, nurses tend to deliver the required nursing care early (NS9)

This was in contrast to older, more stable and independent patients who were used to receiving their medications early.

### 4.2 ANC interventions

Some interventions were reported to be at risk of being delivered early: all students reported the case of medications that were habitually prepared (e.g. by infusion and dilution) and administered early as compared to the specified time. Specifically, while oral medications were more often administered in advance (e.g. at 3 p.m. instead of 4 p.m.), intravenous medications, despite being prepared early, were usually administered at the specified time.

Taking care of basic needs throughout the day was also reported as being delivered before the expected time. Preparing patients for the night was reported as being carried out during the afternoon shifts. Moreover, blood samples were collected by night‐shift nurses who ended their shift at 7 a.m., even if the specified time for the examination was 8 a.m. Furthermore, students reported that venous access was inserted early when patients still needed to be assisted for all their needs and when a second cannula had to be inserted due to phlebitis in the one already present. Sometimes dressing changes were reported as being renewed in advance when, for example, nurses were in the patient's room and wanted to have all aspects under control and “*in order*.” Moreover, asking discharged patients to leave their beds prematurely because of high pressure from the emergency department to admit new patients was also reported as frequent: these patients were required to wait in the dining room or to return home before time. On the other hand, nurses were used to anticipating their arrival in the unit with respect to the beginning of their shift. Before the official start of shifts, nurses were used to performing the first round of patients, reading the nursing records of the newly admitted patients and receiving verbal handovers (Table 4).

### 4.3 ANC antecedents

Five main reasons were reported by students as antecedents of ANC (Table 4):

1. Time‐management issues, where nurses feel the need to manage the large amount of time required by some interventions by starting tasks early in an attempt to finish on time and by adapting less time‐consuming interventions by doing them early to gain time to organize the remaining interventions.
2. The scarcity of nursing resources and the implementation of organization models forcing efficiency in the work processes; ANC is thus aimed at compensating for these shortages.
3. The attempt to be ready for the unexpected, as unpredictable events in clinical conditions or in the number of patients are more easily managed when all scheduled activities are completed.
4. Professional issues as divided into two different aspects: when lack of trust within the nursing staff triggers nurses to perform all interventions without delay and by the end of the shift, and on the other hand, because nurses were reported by students to be proud of performing all activities during their shift as a symbol of responsibility.
5. Some organizational issues triggered by old‐fashioned habits and routines also with respect to the work processes of other healthcare professionals that impose the need to finish some activities early.

### 4.4 ANC consequences

Different outcomes were perceived by students as associated with ANC. Firstly, at the nurses’ level, one outcome was not leaving tasks to colleagues on the next shift, which allowed them to start immediately with their activities and to feel more relaxed. This was reported to trigger emotional outcomes such as feeling gratified, as a result of increased collaboration and solidarity among nurses. At the same time, leaving the unit “*in peace”* was also reported as having an emotional outcome. Ending the shift having completed all activities, without leaving anything to be carried out by colleagues on the next shift, and an appropriate physical space to give accurate handovers were considered signs of professional performance, which increased satisfaction and reduced stress. At the patients’ level, ANC was reported by students as a strategy to prevent missed or delayed care; for this reason, students reported that patients were satisfied when receiving nursing care in advance (Table 4).

## 5 DISCUSSION

To the best of our knowledge, this is the first study that attempts to describe ANC, together with its antecedents and consequences, as experienced by students during their clinical rotations. Prior to recent studies (Bottega & Palese, 2020), ANC was reported mainly through anecdotal evidence as an expression of routines, traditions and rituals of nursing practice, and a potential issue of the theory–practice gap (Bourgault & Upvall, 2019; Rytterstrom et al., 2011; Shoghi et al., 2019), mostly from the perspective of nurses. However, the clinical environment permeates nursing education, enabling students to develop skills (Papastavrou et al., 2016). Therefore, exploring their perceptions of not only MNC but also ANC might support the understanding of how students shape their ability to prioritize in the face of competitive needs and interventions.

Nursing students reported that the phenomenon of ANC is widespread, valued and implicitly considered in clinical practice through examples. Therefore, what has already been reported with regard to MNC, of which nursing students seem to be developing a progressive awareness of (Greenway et al., 2019) that triggers a cognitive dissonance (Bagnasco et al., 2017), appears to be similar in the case of ANC. Moreover, as in the case of MNC (Bagnasco et al., 2017), students reported experiencing a sort of “pragmatic acceptance” where anticipating care is an integral part of the nursing work, as is missed care (Gibbon & Crane, 2018).

In describing their perceptions on ANC, students seem to develop an early awareness during their clinical rotations that focuses on managing the available time and providing a range of interventions, which could be considered one of the main issues for nurses. Based on their narratives, nurses are perceived as always being in a “*hurry*” and trying to move the care plan forward. Given that clinical training also shapes professional values and attitudes (Maranon & Pera, 2015), students are also exposed early to how to challenge the lack of time, an issue that will permeate their professional career. Moreover, given that not all patients are exposed to ANC, student nurses are at risk of seeing and replicating further models of care differentiation that can also have ethical implications with potential consequences of discrimination (Scott et al., 2019).

Students reported that some interventions are more often anticipated, while others are forerunners of future activities. In the former case, for instance, a student reported witnessing – and thus learning – that medications prescribed for different times, but close to each other, were being administered by nurses at the same time with a single administration. In the latter case, waking up patients early in the morning to perform nursing activities was described as an occasion to anticipate other basic nursing care activities (e.g. hygiene). However, several nursing interventions were reported to be anticipated: specifically, those affecting direct care (e.g. medication administration and fundamentals of care) and indirect nursing, with nurses arriving in the unit before the expected time in order to get to know patients in advance and to collect data from handovers. Moreover, some of the reported tasks were independent care activities (e.g. those related to the fundamentals of care), while others were interdependent with physicians (e.g. medication administration), and several others involved an interdependency among other units (e.g. asking patients to leave their beds). All these multidimensional aspects of the ANC phenomenon as perceived by students can stimulate the belief that nurses are situated in the middle of the process of care and are not its leader. This may negatively shape the self‐perception of students as future nurses.

Although students’ complete lack of awareness regarding the reasons for MNC has already been documented, due to their partial involvement in the nurses’ decision‐making processes (Gibbon & Crane, 2018), in the case of ANC they reported several antecedents. According to their perceptions, in the attempt to maximize time management, nurses try to anticipate some activities in order to leave the following care interventions at the bedside free of interferences. Unpacking a process, by allocating a specific sequence to the intervention at a specific time, allows nurses to exert control over time. Moreover, time control has also been reported as a pivotal factor with regard to the time required for each intervention; if time is predictable, nurses perform these activities first, leaving those that require an unpredictable amount of time until after. According to students, nurses also try to cope with the limited resources available at the bedside by anticipating the care required by patients. Despite time limitations, they try to adapt all the activities required in a sort of chain, where inevitably some are performed earlier and others later. Moreover, they reported that nurses tend to be prepared for unpredictable events by ANC because of the possibility of unexpected emergencies. This factor has been reported in the Italian context as also being associated with MNC, in addition to the causes reported in other countries, such as communication issues and human and material resources issues (Bottega & Palese, 2020). In summary, among the different reasons reported, a common origin seems to be the lack of time. This suggests that students should be educated on how to survive effectively by learning early time‐management and priority‐setting skills. However, to date, a lack of studies in the field of nursing students and time management is detected (Oksuz et al., 2018).

On the other hand, at the professional level, students reported that some activities were being anticipated as a result of the lack of trust within the nursing team and as evidence of professional responsibility, suggesting that ANC can shape the acquisition of professional values and attitudes. At the organizational level, a unit's habits and routines, such as professional interdependencies and hierarchies, as well as processes established at the unit level, which proceed without challenge because of beliefs and expectations in the ward, were reported as triggering ANC. While routine is something instrumental and superficial that tends to repeat itself over time (Philpin, 2002), ritual, on the other hand, has a symbolic meaning that persists over time (Rytterstrom et al., 2011). In other words, students seem to attribute ANC to factors associated with the so‐called “*sociology of time*” as the basic mechanism through which human behaviour is organized and regulated – the temporal structure of actions resulting from an implicit agreement of inter‐subjective experience in a given context, which establishes a normative organizational behaviour. Therefore, these factors intertwined with the sociology of time might anticipate, delay or omit some interventions, leading nurses to provide care that is not patient centred (Ghane & Esmaeili, 2020; Greenway et al., 2019; Rytterstrom et al., 2011).

With regard to consequences, ANC interventions seemed to enhance the work environment and nurses’ well‐being, as well as prevent MNC. Students described no negative emotions regarding ANC, contrary to those reported in MNC (Gibbon & Crane, 2018). Therefore, according to the role model expressed by clinical nurses, students seemed to progressively learn how to anticipate nursing care by internalizing working values witnessed in their clinical practice, given the common perception that this is accepted and rewarded by nursing colleagues, the organization and patients.

### 5.1 Limitations

The study is affected by several limitations. Firstly, we involved only four nursing programmes, and all were located in Northern Italy; as a result of federalization of the healthcare system at the regional level (France et al., 2005), variations in education as well as in clinical practice patterns are documented (Palese et al., 2019). Therefore, further investigation of the phenomenon in other contexts is suggested. Secondly, we recruited students at different levels of their nursing education who had a different number of clinical rotations (from none for the first‐year students to several for the third‐year students); thus, the participants had different degrees of ability in establishing the peculiarities of the ANC phenomenon by comparing their experiences over the years in different contexts. Regarding antecedents, nursing students provided insights according to their clinical experience gained mainly in acute care contexts. Therefore, futures studies should investigate if the same causes are reported by nursing students in other contexts, such as long‐term care and community.

## 6 IMPLICATIONS FOR NURSING EDUCATION AND PRACTICE

The use of a qualitative‐descriptive approach elicited crucial information on ANC as perceived by nursing students. The findings can be useful at the undergraduate nursing level to raise awareness of the phenomenon and to give insights regarding how to develop important competences, such as time‐management and priority‐setting skills, that should be integrated in practice. Both positive and negative meanings of ANC seem to emerge as two sides of the same coin. While some patients have been reported to be at risk of receiving an intervention prematurely, as in the case of medication administration, ANC was also perceived as a sign of both initiative and proactivity by nurses and thus at merit to be mirrored by nursing students, which might be rewarded in the evaluation at the end of the clinical rotation. Helping students to differentiate between anticipated and proactive nursing care, with the latter being the skill to predict needs in advance, is crucial.

ANC is a paradigmatic example of practice from which students learn values, beliefs and attitudes towards the nurse's role; their position in the clinical scenario; and their position in relation to other professions. Being immersed in a context that values the capacity to deliver interventions irrespectively at the correct time, without openly discussing the reasons, implications and alternative solutions, might be replicated by future generations using the same patterns of priority‐setting and time‐management strategies. Moreover, the perception of professional hierarchies as a cause of ANC might reinforce among undergraduate students the view that the profession is not fully independent; in this regard, the implications of academic attrition should be considered. Therefore, supporting students and promoting their critical awareness are important through a discussion of not only the intervention's appropriateness but also regarding its adequate time of delivery.

## 7 CONCLUSION

Alongside the widely reported phenomenon of MNC, nursing students perceived that ANC is common and reflects required interventions that are delivered in advance of the expected and/or specified time. Different independent, dependent and interdependent nursing interventions emerged and are usually performed in advance, mainly with respect to stable and older patients with low and high functional dependency, suggesting that students are thought to differentiate patients as meriting reception of the care earlier than expected. This multidimensional phenomenon is perceived by nursing students as a reaction to the lack of resources and to professional and organizational issues, which therefore shape their priority‐setting skills around these factors. The reported consequences, which are mainly positive from the perspective of nursing students, might strengthen their belief that ANC is acceptable and an effective care measure — an attitude they might replicate once they become newly graduated nurses. Future research is strongly recommended to accumulate evidence in the field, aiming to quantitatively establish the extent of the phenomenon, as well as to identify how ANC can affect nursing education and, ultimately, the quality of nursing care.

## CONFLICT OF INTEREST

No conflict of interest has been declared by the authors.

## AUTHOR CONTRIBUTIONS

AP and MB contributed to the study concept and design. LL and AP collected data. AP and LL performed the data analysis. LL, AP and MD analyzed and drafted the manuscript. All authors critically reviewed and approved the final version of the manuscript.

## Supporting information

Table S1

## Data Availability

Data available on request from the authors.
