# Supplementary material for: Anticipated nursing care as perceived by nursing students: Findings from a qualitative study
Source: Nurs Open. 2021 May 3;8(6):3373–83. doi: 10.1002/nop2.883 (PMC8510745; doi:10.1002/nop2.883)
Supplement: Supplementary file 1 — Table S1 [file NOP2-8-3373-s001.docx]

**Supplementary table 1** Analysis of the study conduction according to the COnsolidated criteria for REporting Qualitative research (Tong et al. 2007)

| **No. Item** | **Guide questions/description** | **Reported on Page No.** |
| --- | --- | --- |
| **Domain 1:** **Research team and reﬂexivity** | |  |
| **Personal Characteristics** | |  |
| 1. Interviewer/facilitator | Which author/s conducted the interview or focus group? | LL (see author) and AP (see author) |
| 2. Credentials | What were the researcher’s credentials? E.g. PhD, MD | LL is a Registered Nurse, Bachelor Nursing Degree; AP is a PhD Nurse |
| 3. Occupation | What was their occupation at the time of the study? | LL was a nursing student at the time of the study; AP was an Associate Professor of Nursing Science |
| 4. Gender | Was the researcher male or female? | Both were Female |
| 5. Experience and training | What experience or training did the researcher have? | LL was supervised by AP; AP was an expert researcher |
| **Relationship with participants** | |  |
| 6. Relationship established | Was a relationship established prior to study commencement? | None; no personal contacts (e.g. friendship) was established prior the study |
| 7. Participant knowledge of the interviewer | What did the participants know about the researcher? | They were introduced to the main aim of the study in the initial contacts |
| 8. Interviewer characteristics | What characteristics were reported about the interviewer or facilitator? | A few data have been reported; only the academic position and the intent of the study have been reported |
| **Domain 2: Study design** | |  |
| **Theoretical framework** | |  |
| 9. Methodological orientation and Theory | What methodological orientation was stated to underpin the study? | There was a undertaken a descriptive qualitative study |
| **Participant selection** | |  |
| 10. Sampling | How were participants selected? | There was used a purposeful sample |
| 11. Method of approach | How were participants approached? | There was contacted 18 nursing students |
| 12. Sample size | How many participants were in the study? | A total of 16 nursing students agreed to participate |
| 13. Non-participation | How many people refused to participate and why? | Two nursing students refused to partecipate |
| **Setting** | |  |
| 14. Setting of data collection | Where was the data collected? e.g. home, clinic, workplace | Participants were interviewed in different places: (a) room of the university; (b) public contexts (library, pub); (c) via skype (two participants), according to the preferences of the participants |
| 15. Presence of non-participants | Was anyone else present besides the participants and researchers? | There was present AP (see authors), with the intent to supervise the conduction of the interview and its quality |
| 16. Description of sample | What are the important characteristics of the sample? | The important characteristics of the sample are reported in Table 1 |
| **Data collection** | |  |
| 17. Interview guide | Were questions, prompts, guides provided by the authors? Was it pilot tested? | The interview guide was developed and piloted, as reported in the data collection methods. In the Table 2, the questions have been reported |
| 18. Repeat interviews | Were repeat interviews carried out? If yes, how many? | The interviews were not repeated to not burden students |
| 19. Audio/visual recording | Did researchers use audio/visual recording to collect the data? | The interviews were all audio-recorded after having obtained the written informed consent |
| 20. Field notes | Were ﬁeld notes made during and/or after the interviews? | According to the descriptive nature of the study, no in-the field notes have been collected |
| 21. Duration | What was the duration of the interviews or focus group? | In the Table 2, the duration (minutes, seconds) of each interview has been reported |
| 22. Data saturation | Was data saturation discussed? | The saturation of the data has been discussed independently by two researchers |
| 23. Transcripts returned | Were transcripts returned to participants for comment and/or correction? | Two transcribed interviews have been reported to participants for comments and corrections, making the first member to check with them (Sandelowski 2010) thus ensuring also the quality of the process |
| **Domain 3: Analysis and ﬁndings** | |  |
| **Data analysis** | |  |
| 24. Number of data coders | How many data coders coded the data? | Two researchers coded the data (LL, AP, in the first stage; then all authors) |
| 25. Coding tree description | Did authors provide a description of the coding tree? | The coding tree is fully reported in Table 4 |
| 26. Derivation of themes | Were themes identiﬁed in advance or derived from the data? | The themes were derived from the data and organized in (a) the phenomenon of ANC as lived during their clinical rotations, (b) its antecedents, and (c) its consequences according to nursing students |
| 27. Software | What software, if applicable, was used to manage the data? | N/A |
| 28. Participant checking | Did participants provide feedback on the ﬁndings? | Two transcribed interviews have been reported to participants for comments and corrections, making the first member to check with them (Sandelowski 2010) thus ensuring also the quality of the process |
| **Reporting** | |  |
| 29. Quotations presented | Were participant quotations presented to illustrate the themes/ﬁndings? | The quotes have been fully reported in Table 4 |
| 30. Data and ﬁndings consistent | Was there consistency between the data presented and the ﬁndings? | The Table 3 reports strategies adopted to ensure consistency |
| 31. Clarity of major themes | Were major themes clearly presented in the findings? | The Table 4 and the findings section report the major themes |
| 32. Clarity of minor themes | Is there a description of diverse cases or discussion of minor themes? | No diverse cases have emerged |

*N/A* Not applicable
